# miR-188-3p-targeted regulation of ATG7 affects cell autophagy in patients with nonobstructive azoospermia

**DOI:** 10.1186/s12958-022-00951-0

**Published:** 2022-06-16

**Authors:** Yuan Wang, Cheng-Cheng Tian, Yun-Yun Jiao, Min-Rui Liu, Xue-Shan Ma, Hai-Xia Jin, Ying-Chun Su, Xiang-Yang Zhang, Wen-Bin Niu, Gui-Don Yao, Wen-Yan Song

**Affiliations:** 1 Center for Reproductive Medicine, The First Affiliated Hospital of Zhengzhou University, Zhengzhou, 450052 China; 2 Department of Reproductive Medicine, Nanyang Central Hospital, Nanyang, 473000 China; 3 Department of Reproductive Medicine, Zhengzhou Maternal and Child Health Hospital, Zhengzhou, China

**Keywords:** Nonobstructive azoospermia, miR-188-3p, Autophagy, ATG7

## Abstract

**Background:**

Nonobstructive azoospermia (NOA) is one of the most difficult forms of male infertility to treat, and its pathogenesis is still unclear. miRNAs can regulate autophagy by affecting their target gene expression. Our previous study found that miR-188-3p expression in NOA patients was low. There are potential binding sites between the autophagy gene ATG7 and miR-188-3p. This study aimed to verify the binding site between miR-188-3p and ATG7 and whether miR-188-3p affects autophagy and participates in NOA by regulating ATG7 to influence the autophagy marker genes LC3 and Beclin-1.

**Methods:**

Testicular tissue from 16 NOA patients and 16 patients with normal spermatogenesis and 5 cases in each group of pathological sections were collected. High-throughput sequencing was performed to detect mRNA expression differences. Quantitative real-time polymerase chain reaction (qRT-PCR), Western blotting, immunohistochemical staining and immunofluorescence were used to detect protein localization and expression. Autophagosome changes were detected by electron microscopy. The targeting relationship between miR-188-3p and ATG7 was confirmed by a luciferase assay.

**Results:**

ATG7 protein was localized in the cytoplasm of spermatogenic cells at all levels, and the ATG7 gene (*p* = 0.019) and protein (*p* = 0.000) were more highly expressed in the NOA group. ATG7 expression after overexpression/inhibition of miR-188-3p was significantly lower (*p* = 0.029)/higher (*p* = 0.021) than in the control group. After overexpression of miR-188-3p, the ATG7 3'UTR-WT luciferase activity was impeded (*p* = 0.004), while the ATG7 3'UTR-MUT luciferase activity showed no significant difference (*p* = 0.46). LC3 (*p* = 0.023) and Beclin-1 (*p* = 0.041) expression in the NOA group was significantly higher. LC3 and Beclin-1 gene expression after miR-188-3p overexpression/inhibition was significantly lower (*p* = 0.010 and 0.024, respectively) and higher (*p* = 0.024 and 0.049, respectively). LC3 punctate aggregation in the cytoplasm decreased after overexpression of miR-188-3p, while the LC3 punctate aggregation in the miR-188-3p inhibitor group was higher. The number of autophagosomes in the miR-188-3p mimic group was lower than the number of autophagosomes in the mimic NC group.

**Conclusions:**

LC3 and Beclin-1 were more highly expressed in NOA testes and negatively correlated with the expression of miR-188-3p, suggesting that miR-188-3p may be involved in the process of autophagy in NOA. miR-188-3p may regulate its target gene ATG7 to participate in autophagy anDual luciferase experiment

d affect the development of NOA.

**Supplementary Information:**

The online version contains supplementary material available at 10.1186/s12958-022-00951-0.

## Introduction

Nonobstructive azoospermia (NOA) can cause testicular spermatogenesis dysfunction, resulting in the inability to produce sperm or producing a very small amount of sperm [1]. NOA is a kind of heterogeneous disease caused by complex genetics, including chromosomal abnormalities, gene mutations and environmental factors. Previous research on NOA focused on chromosomal abnormalities [2], which explained only approximately 25% of azoospermia cases. However, the aetiology of nonchromosomal NOA is complex, and its pathogenesis is unclear. In recent years, many new technologies have been applied to the treatment of NOA, but the overall cure rate is still low. Therefore, an in-depth study of the molecular mechanisms affecting the number of spermatozoa produced in NOA patients will help to identify new molecular markers for NOA diagnosis and gene therapy targets.

Early research indicated that some miRNAs can regulate the expression of target genes and participate in a variety of physiological and pathological processes [3]. Animal experiments have shown that downregulated miR-188-3p expression leads to abnormal germ cell development and causes sperm formation disorders [4]. Additionally, our previous study also found that miR-188-3p regulates the target gene MLH1 and contributes to the occurrence of NOA by promoting spermatogenic cell apoptosis [5]. These results suggest that miR-188-3p is related to spermatogenesis.

In recent years, an increasing number of studies have indicated that miRNAs take part in the regulation of autophagy by regulating the expression of their target genes [6]. Autophagy, which participates in the development of various diseases, is a process in which damaged organelles and macromolecules are degraded by lysosomes under the regulation of autophagy-related genes. ATG7 is an autophagy-related gene that is crucial for the formation of autophagy coupling systems [7]. Studies found that ATG7 gene knockout mice had acrosomal development defects, and the sperm shape was similar to the sperm shape of human round head sperm [8]. In addition, our previous high-throughput sequencing data revealed that miR-188-3p and ATG7 have binding sites. However, further studies are needed to confirm whether miR-188-3p and ATG7 have a direct targeting regulatory relationship to affect spermatogenesis.

## Materials and methods

### Sample collection

Testicular tissues were collected from NOA patients (16 cases) and patients with normal spermatogenesis (16 cases) in the Reproductive Medicine Center of the First Affiliated Hospital of Zhengzhou University from October 2016 to October 2019. There were 5 cases in each group of pathological sections in the pathology department of our hospital. All patients enrolled in the study were aged between 22 and 43 years. Testicular tissue of NOA was obtained from testicular puncture of NOA patients undergoing intracytoplasmic sperm injection (ICSI) at the Reproductive Medicine Center, and testicular tissue of normal spermatogenic function was obtained from testicular puncture of male patients due to difficult sperm retrieval failure on the day of oocyte retrieval. The study was approved by the ethics committee of the First Affiliated Hospital of Zhengzhou University (2019-KY-78). All the participants signed informed consent forms.

According to the guidelines of the World Health Organization in 2010, when: ① there are no sperm in the semen analysis three times after centrifugation and inverted microscope screening, ② B-ultrasound examination shows that the vas deferens and epididymis are unobstructed or nonexistent, ③ testicular biopsy shows that there are no sperm or only a small amount of sperm, or spermatogenic cells are completely missing, and spermatogenic epithelium is composed only of Sertoli cells, the condition can be regarded as NOA [9]. Male patients with chromosomal abnormalities and patients with a clear history of exposure to toxicants and radiation were excluded.

The groups in this experiment were as follows. High-throughput sequencing experiments were divided into the NOA group and the control group, with 3 cases of testis tissue in each group. Quantitative real-time polymerase chain reaction (qRT-PCR) and Western blot experiments were also performed in the NOA group and the control group, with 13 cases in each group (the clinical information is summarized in Table 1). Immunohistochemical experiments using pathological sections included 5 cases in the NOA group and 5 cases in the control group (the clinical information is summarized in Table 2). Cell experiments were performed in a human testicular cancer cell line (NTERA-2, NT-2). According to whether overexpression/inhibition was performed, cells were divided into the miR-188-3p mimic group/miR-188-3p mimic NC group and miR-188-3p inhibitor group/miR-188-3p inhibitor NC group.

**Table 1:** The clinical information of patients whose testis tissue collected in the reproductive medicine center(x ± s)

Index	NOA group (*n* = 16)	Control group (*n* = 16)	*P-*value
Age (years)	27.25 ± 3.61	29.50 ± 5.03	0.233
FSH (IU/L)	21.30 ± 6.86*	5.13 ± 2.94	0.000
LH (IU/L)	10.44 ± 4.34*	4.64 ± 1.92	0.000
T (ng/ml)	3.27 ± 2.39	4.60 ± 2.12	0.053
PRL (ng/ml)	12.65 ± 4.82	9.91 ± 4.26	0.254
E2 (pg/ml)	20.59 ± 13.68	26.55 ± 9.10	0.143
P (ng/ml)	0.50 ± 0.27	3.11 ± 9.31	0.877
Left testis volume (ml)	7.44 ± 1.93*	13.44 ± 3.85	0.000
Right testis volume (ml)	8.00 ± 2.66*	13.44 ± 3.85	0.000
Number of patients with abnormal secondary sexual characteristics no./total no. (%)	0/16 (0)	0/16 (0)	1.000
Number of patients with varicocele no./total no. (%)	0/16 (0)	0/16 (0)	1.000
Number of patients with genital abnormalities no./total no. (%)	0/16 (0)	0/16 (0)	1.000
Number of patients with abnormal karyotype no./total no. (%)	0/16 (0)	0/16 (0)	1.000
Number of patients with Y chromosome microdeletions no./total no. (%)	0/16 (0)	0/16 (0)	1.000

Plus–minus values are means ± SD. To convert values for estradiol to picomoles per liter, multiply by 3.671. To convert values for testosterone to nanomoles per liter, multiply by 3.467

Explanation for the relevant abbreviations:

*FSH* means Follicle-stimulating hormone, *LH* means Luteinizing hormone, *T* means Total testosterone, *PRL* means prolactin, *E2* means Estradiol and *P* means Prog

^*^Statistically significant difference compared with control group (*P* < 0.05)

**Table 2:** The clinical information of patients whose pathological sections in the pathology department(x ± s)

Index	NOA group (*n* = 5)	Control group (*n* = 5)	*P-*value
Age (years)	31.20 ± 4.87	28.40 ± 8.79	0.292
FSH (IU/L)	21.53 ± 9.67*	6.46 ± 1.25	0.009
LH (IU/L)	12.00 ± 5.79*	3.59 ± 0.52	0.009
T (ng/ml)	2.99 ± 0.40	4.36 ± 1.26	0.075
PRL (ng/ml)	10.97 ± 3.05	8.89 ± 1.50	0.116
E2 (pg/ml)	21.13 ± 6.4	30.16 ± 7.32	0.076
P (ng/ml)	0.25 ± 0.11	0.42 ± 0.20	0.059
Left testis volume (ml)	6.40 ± 1.67*	12.80 ± 2.95	0.014
Right testis volume (ml)	6.40 ± 1.67*	12.80 ± 2.95	0.014
Number of patients with abnormal secondary sexual characteristics no./total no. (%)	0/5 (0)	0/5 (0)	1.000
Number of patients with varicocele no./total no. (%)	0/5 (0)	0/5 (0)	1.000
Number of patients with genital abnormalities no./total no. (%)	0/5 (0)	0/5 (0)	1.000
Number of patients with abnormal karyotype no./total no. (%)	0/5 (0)	0/5 (0)	1.000
Number of patients with Y chromosome microdeletions no./total no. (%)	0/5 (0)	0/5 (0)	1.000

Plus–minus values are means ± SD. To convert values for estradiol to picomoles per liter, multiply by 3.671. To convert values for testosterone to nanomoles per liter, multiply by 3.467

Explanation for the relevant abbreviations:

*FSH* means Follicle-stimulating hormone, *LH* means Luteinizing hormone, *T* means Total testosterone, *PRL* means prolactin, *E2* means Estradiol and *P* means Prog

^*^Statistically significant difference compared with control group (*P* < 0.05)

### High-throughput sequencing of testicular tissue

The testicular tissue was fully ground in a clean mortar with an appropriate amount of liquid nitrogen. After the grinding was complete, TRIzol reagent (Invitrogen, USA) was added to extract total RNA from testicular tissue (according to the instructions provided by the manufacturer). The sequencing was completed by mRNA enrichment, rRNA removal, end flattening, 3’ end plus A, splicing, PCR amplification, library quality control, library standardization and enrichment. Cluster generation and first-way sequencing primer hybridization were completed on the cBot of the Illumina sequencer according to the cBot User Guide. Sequencing reagents were prepared according to the Illumina User Guide, and the flow cell with the cluster was uploaded to the machine. The PAIRED-END program was selected for double-ended sequencing, and the entire sequencing process was controlled by the DATA COLLECTION software provided by Illumina, with real-time data analysis. Quality control analysis criteria: The sequencing result analysis data volume of each sample was approximately 10 G, and the proportion of each base mass greater than 20 (Q20) was not less than 90%.

### Cellular transfection

Cells were inoculated at a density of approximately 1.2 × 10^6^ cells per well, with cell confluence of 70%-80%. Preparation of liquid A: Five microlitres of miR-188-3p mimics/control mimics and inhibitors/suppressors at a concentration of 20 µM were added to 250 µl serum-free Opti-minimal essential medium (MEM), mixed well, and incubated at 23 °C for 5 min. Preparation of liquid B: Liposomal Lipo2000 was mixed before being used, and 5 µl of Lipo2000 was added to 250 µl serum-free Opti-MEM (according to Opti-MEM: Lipo2000 = 50:1), mixed well, and incubated at 23 °C for 5 min. The two liquids were mixed well and incubated at 23 ºC for 20 min. The mixture was slowly added to a 6-well plate cell culture dish and incubated at 37 °C and 5% CO2 for 4 ~ 6 h. Then, the serum-free medium was replaced with Dulbecco’s modified Eagle medium (DMEM) containing 10% serum.

### qRT-PCR

TRIzol reagent was used for extracting total RNA; the optical density (OD) value (OD260/OD280) and RNA concentration of the extracted RNA were determined. mRNAs were synthesized using primers from the kit, whereas for miRNAs, cDNA synthesis was performed using specific reverse transcription primers. The specific primers for miR-188-3p were as follows:

5 ‘-GTCGTATCCAGTGCAGGGTCCGAGGTATTCGCACTGGATACGACTGCAAA-3’. The reverse transcription (RT) reaction solution was prepared by adding total RNA at 1 ~ 2 µg on ice (Supplementary Table SI). Two parallel control wells were set up per sample, and the experiment was repeated at least three times. The relative expression of the genes was calculated according to the Eq. 2^−ΔΔCt^, and the endogenous control for microtubule-associated protein 1 light chain 3 (LC3), Beclin-1 and ATG7 was glyceraldehyde-3-phosphate dehydrogenase (GAPDH), while the endogenous control for miRNA-188-3p was U6. The specific primers were designed by Premier 3.0 and are shown in Supplementary Table II. The quantitative real-time PCR configuration is shown in Supplementary Table SIII. The set parameters for the PCR conditions are shown in Supplementary Table SIV.

### Western blot

Total protein was extracted from spermatogenic cells of testicular tissue and NTERA-2 cells. Western blotting for ATG7, LC3, and Beclin-1 was performed as described in our previous work ^8^. The membrane was incubated with antiATG7 antibody (1:1000; Abcam, Cambridge, UK), antiLC3 antibody (1:1000; Abcam), antiBeclin-1 antibody (1:1000; Abcam) and antiβ-actin antibody (1:1000; Abcam) overnight at 4 ºC and incubated with secondary antibodies (1:1000; Sanying Biotechnology, Wuhan, China) for 2 h at 23 °C.

### Dual luciferase experiment

ATG7 3'UTR: 5'….. AUGGGUGAGGGUGGGAC…0.3'.

miR-188-3p: 3' ACGUUUGGGACGUACACCCUC 5'.

According to the prediction results, the ATG7 3'-UTR dual luciferase reporter wild-type (WT) and mutant (MUT) plasmids were constructed as follows:

- ATG7 3'UTR-WT:
  5'-… CCTGTGGGGGCCCTGGGCATGGGTGAGGGTGGGAC…-3'
- ATG7 3'UTR-MUT:
  5'-… CCTGTGGGGGCCCTGGGCATGGGTGAGGCACCCTC…-3'

The transfected cells were divided into four groups: ① cells transfected with miR-188-3p mimic at a final concentration of 50 nM and 400 ng ATG7 3'UTR-WT plasmid per well in a 24-well plate; ② cells transfected with miR-188-3p mimic NC at a final concentration of 50 nM and 400 ng ATG7 3'UTR-WT plasmid per well in a 24-well plate; ③ cells transfected with miR-188-3p mimic at a final concentration of 50 nM and 400 ng ATG7 3'UTR-MUT plasmid per well in a 24-well plate; and ④ cells transfected with miR-188-3p mimic NC at a final concentration of 50 nM and 400 ng ATG7 3'UTR-MUT plasmid per well in 24-well plates. After 48 h of transfection, the cell culture medium was discarded. Subsequently, 100 μl of 1 × phospholipase B (PLB) per well was added to the cells in a 24-well plate, and the cells were lysed at 23 ºC for 15 min. The sample was centrifuged at 12,000 rpm for 10 min at 4 ºC. The centrifuged supernatant was added to a 96-well plate at 10 µl per well. Each sample required 2 parallel wells for a total of 3 wells. Each well sample was increased to 50 µl/well by adding 40 µl 1 × PLB (to avoid sample evaporation affecting the experimental results). Then, 100 µl LAR II was added to each well to quantify firefly luciferase activity, and the absorbance value at 480 nm was measured. Subsequently, 100 µl of 1 × Stop&Glo® Reagent working solution was added to quench the fluorescence of LARII and simultaneously excite Renilla luciferase fluorescence (internal reference fluorescence). After the treatment, the absorbance value at 480 nm was measured again. The ratio of the two was the relative luciferase activity.

### Immunofluorescence staining analysis

The cells were fixed with 4% paraformaldehyde (PFA) for 15 min and then washed with phosphate-buffered saline (PBS) three times for 3–5 min each time. The sample was placed in a humidified box containing 0.5% Triton X-100/PBS at 23 ºC for 20 min and washed 3 times with PBS. Subsequently, the samples were incubated with antiLC3 antibody (1:100), placed in a wet box, incubated overnight at 4 ºC, and washed 3 times with PBS. An antifluorescence quencher was used to mount the slide, and images were collected under a confocal fluorescence inverted microscope.

### Electron microscope experiment

After 48 h of transfection, the discarded cell medium was quickly added to 2.5% glutaraldehyde electron microscopy fixing solution without rinsing, and the cells were scraped off gently to collect the cells into the centrifuge tube. The cells were then collected by centrifugation, added to new electron microscope fixative solution, fixed at 23 ºC for 2 h, and stored at 4 ºC. After fixing with 1% osmium acid for 1 to 2 h, specimens were incubated in 50%, 70%, 80%, and 90% acetone for 5 to 10 min and washed in 100% acetone three times, each time for 10 to 15 min. Subsequently, the samples were incubated in a mixed solution of 100% dehydrating agent and an equal amount of embedding agent at 23 ºC for 30 min. Then, these samples were placed in pure embedding agent overnight and embedded. The samples were sliced at 70 nm by an ultrathin microtome and double-stained with 3% uranyl acetate-lead citrate. Finally, two sets of samples were placed under a transmission microscope to observe and film.

### Statistical analysis

SPSS 21.0 software was used for statistical analyses. Measurement data are expressed as the mean ± standard deviation (mean ± SD), and the mean between the two groups was compared and analysed by t-test. The test standard was α = 0.05, and P < 0.05 indicated statistical significance. GraphPad Prism 5.0 software was used for mapping analysis.

## Results

### The results of high-throughput sequencing and expression and localization of the ATG7 gene in the testis

**Fig. 1:**
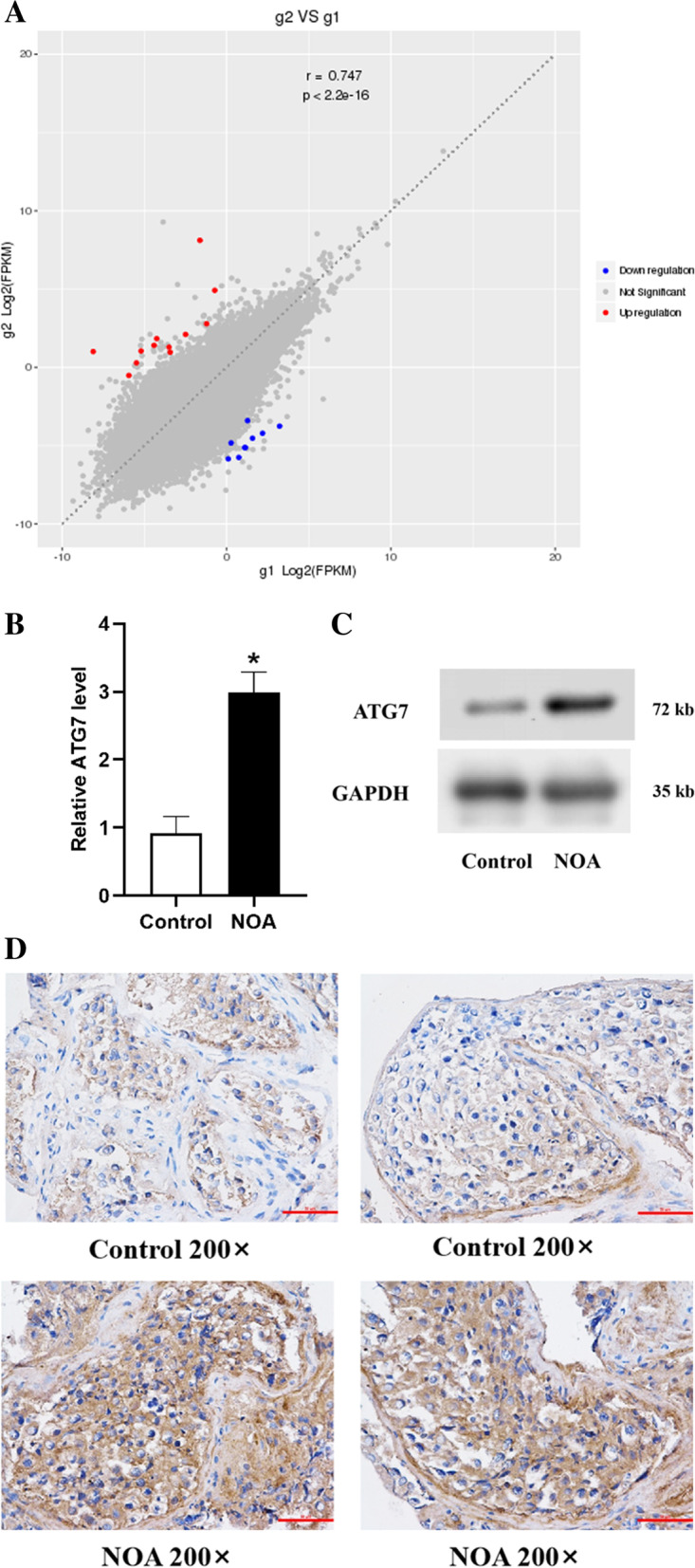
The results of high-throughput sequencing and expression and localization of the ATG7 gene in the testis. **A** The results of differentially expressed genes in high-throughput sequencing analysis between NOA patients and patients with normal spermatogenesis (*n* = 3). **B-C** In the patients with NOA, the expression levels of the ATG7 gene (**B**) and protein (**C**) were higher than the expression levels of the ATG7 gene in the control group, as detected by qRT-PCR and Western blotting. **D** ATG7 protein staining was brownish yellow and was localized in the cytoplasm of spermatogenic cells at all levels. *Statistically significant difference compared with the control group (*P* < 0.05)

Three NOA patients and 3 patients with normal spermatogenesis were used for high-throughput sequencing analysis. The obtained P value was subjected to multiple hypothesis testing and corrected by controlling the false discovery rate (FDR). The Q-value is the corrected P value. The screening conditions for differentially expressed genes were Q-value ≤ 0.05 and fold-change ≥ 2. A total of 109 differentially expressed mRNAs were found (of which 93 mRNAs were upregulated and 16 mRNAs were downregulated) (Fig. 1A). According to bioinformatics analysis and prediction, there were 5 mRNAs with potential binding sites for miR-188-3p. Among them, ATG7 is a key protein for autophagy.

qRT-PCR results showed that ATG7 expression in testicular samples of individuals with NOA was prominently higher (*P* = 0.019) (Fig. 1B). The Western blot results were consistent with the qRT-PCR results (Fig. 1C). The ATG7 protein was observed to be localized mainly in the cytoplasm of spermatogenic cells through immunohistochemistry. ATG7 protein staining was brownish yellow and was localized in the cytoplasm of spermatogenic cells at all levels, and its expression was detected both in NOA and in normal fertility testicular tissues (Fig. 1D). Moreover, the ATG7 protein level in NOA testes was dramatically elevated compared with the ATG7 protein level in normal testes, as detected by integral optical density (IOD) analysis (*P* = 0.000) (Table 3).

**Table 3:** IOD analysis of ATG7 protein expression in testis tissues of each group

Group	n	IOD	*P*-value
Control group	20	47.070 ± 4.7515	0.000
NOA group	20	105.120 ± 8.0311	

### The effect of miR-188-3p overexpression or inhibition on the expression level of ATG7 and the verification of their interaction sites

**Fig. 2:**
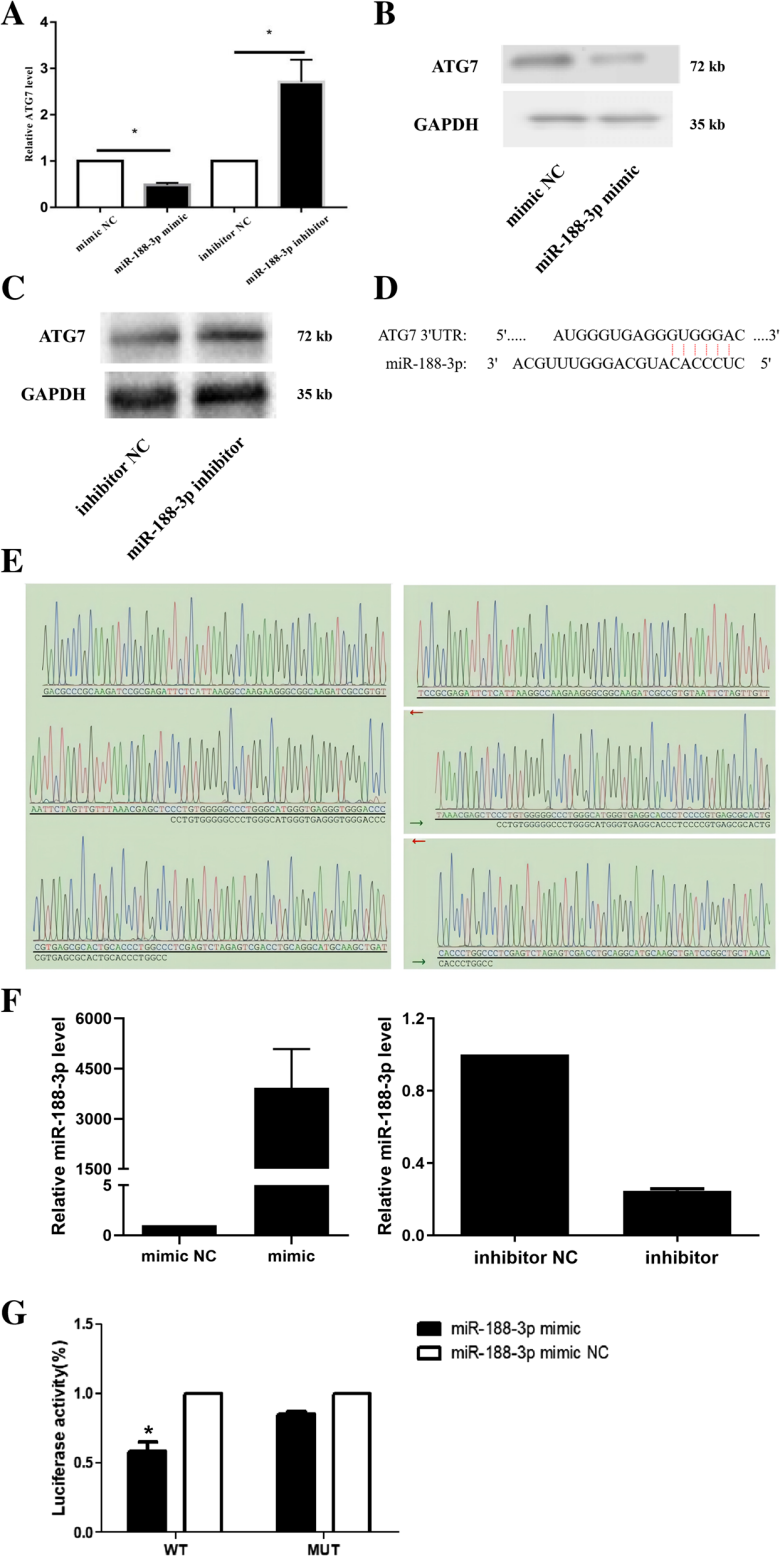
The effect of miR-188-3p overexpression or inhibition on ATG7 expression and the verification of their interaction sites. **A** ATG7 gene expression after overexpression/inhibition of miR-188-3p was significantly lower/higher than the ATG7 gene expression in the control group. **B** The expression of ATG7 protein in the miR-188-3p mimic group was lower than the expression of ATG7 protein in the control group, as detected by Western blot. **C** The expression of ATG7 protein in the miR-188-3p inhibitor group was higher, as detected by Western blot. **D** The mutual binding sites between miR-188-3p and ATG7 were predicted. **E** The ATG7 3'-UTR wild-type (WT) plasmids and mutant (MUT) plasmids were constructed and sequenced as dual luciferase reporter. **F** miR-188-3p expression after miR-188-3p overexpression/inhibition was significantly higher/lower than the miR-188-3p expression in the control group. **G** The luciferase activity of ATG7 3'-UTR WT was lower in the miR-188-3p mimic group than in the miR-188-3p mimic NC group. There was no significant difference in MUT luciferase activity between the two groups. *Statistically significant difference compared with the control group (*P* < 0.05)

NT-2 cells were transfected with miR-188-3p mimic/mimic NC and miR-188-3p inhibitor/inhibitor NC through cell transfection technology. As expected, the ATG7 gene expression level was decreased markedly in the miR-188-3p mimic group compared with the miR-188-3p mimic NC group (*P* = 0.029) (Fig. 2A). The expression of ATG7 protein in the miR-188-3p overexpression samples was lower, as detected by Western blot (Fig. 2B). After miR-188-3p was suppressed, the ATG7 (*P* = 0.021) gene and protein expression levels were considerably higher (Fig. 2A, 2C).

The mutual binding sites were predicted between miR-188-3p and ATG7 according to the analysis of the potential targeting relationship between the two (Fig. 2D). Furthermore, the ATG7 3'-UTR wild-type (WT) plasmids and mutant (MUT) plasmids were constructed and sequenced according to the prediction results as a dual luciferase reporter (Fig. 2E). The overexpression (*P* = 0.028)/inhibition (*P* = 0.000) of miR-188-3p was efficiently checked by qRT-PCR (Fig. 2F). Further examination of dual luciferase showed that the luciferase activity of ATG7 3'-UTR WT was lower in the miR-188-3p mimic group than in the miR-188-3p mimic NC group (*P* = 0.004). However, there was no significant difference in the luciferase activity of the MUT reporter between the two groups (*P* = 0.46) (Fig. 2G).

**Fig. 3:**
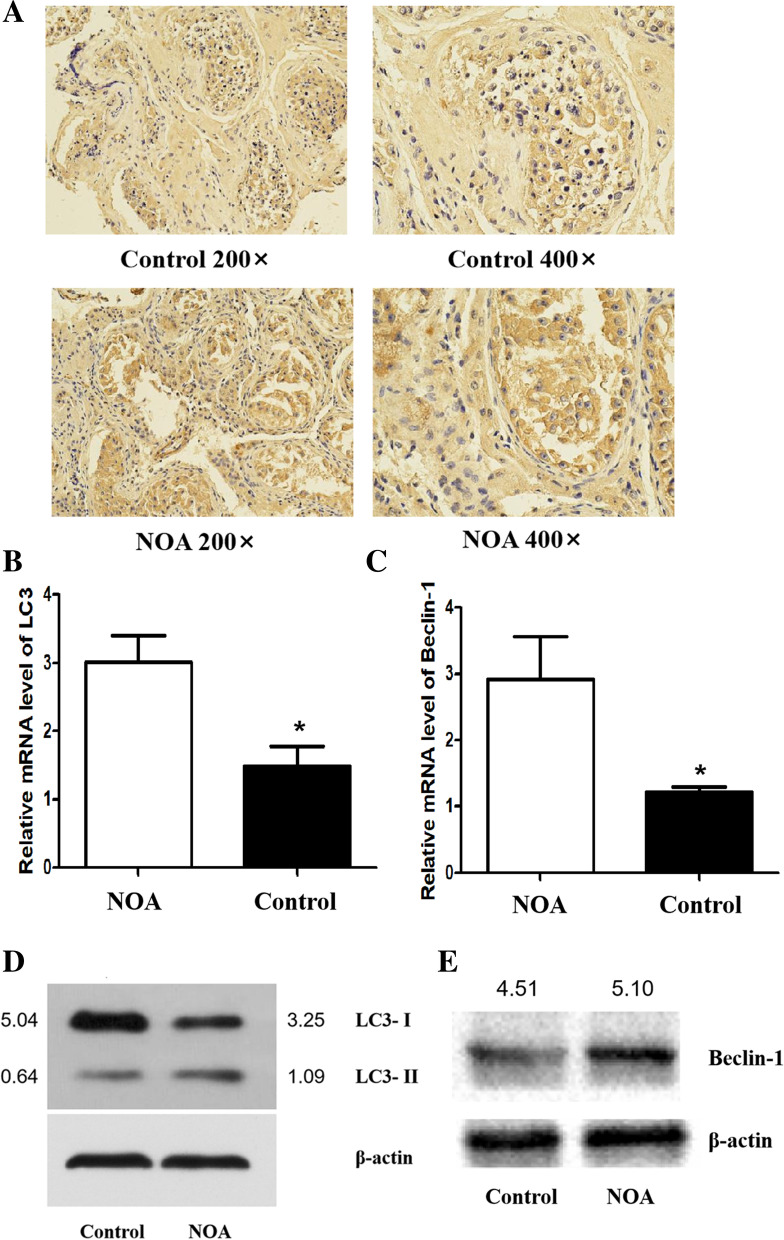
The expression of LC3 and Beclin-1 and the localization of LC3 in testicular tissue. **The** LC3 protein was localized mainly in the cytoplasm of spermatogenic cells at all levels, showing a brownish yellow colour. The LC3 protein level in NOA patients was elevated in comparison with the LC3 protein level in the control group. **B-C** LC3 (**B**) and Beclin-1 (**C**) gene expression levels in the NOA group were enhanced. **D-E** LC3 (**D**) and Beclin-1 (**E**) protein expression levels in the NOA group were higher than the levels in the control group. *Statistically significant difference compared with the control group (*P* < 0.05)

As demonstrated in Fig. 3A and Table 3, LC3 protein was localized mainly in the cytoplasm of spermatogenic cells at all levels, and its staining was brownish yellow. The LC3 protein level in NOA patients was elevated in comparison with the LC3 protein level in the control group, as detected by IOD analysis of immunohistochemical experiments (*P* = 0.009) (Table 4). Moreover, LC3 (*P* = 0.023) and Beclin-1 (*P* = 0.041) expression levels in the NOA group were enhanced (Fig. 3B, 3C). Consistent with the gene expression, the protein levels of the two in the NOA group were higher (Fig. 3D, 3E). In addition, the ratio of autophagosomal membrane type LC3 (LC3-II)/cytoplasmic LC3 (LC3-I) in the NOA group was also elevated (Fig. 3D).

**Table 4:** IOD analysis of LC3 protein expression in testis tissues of each group

Group	n	IOD	*P*
Control group	5	39.478 ± 4.473	0.009
NOA group	5	76.241 ± 4.213	

### The effect of miR-188-3p overexpression or inhibition on autophagy

**Fig. 4:**
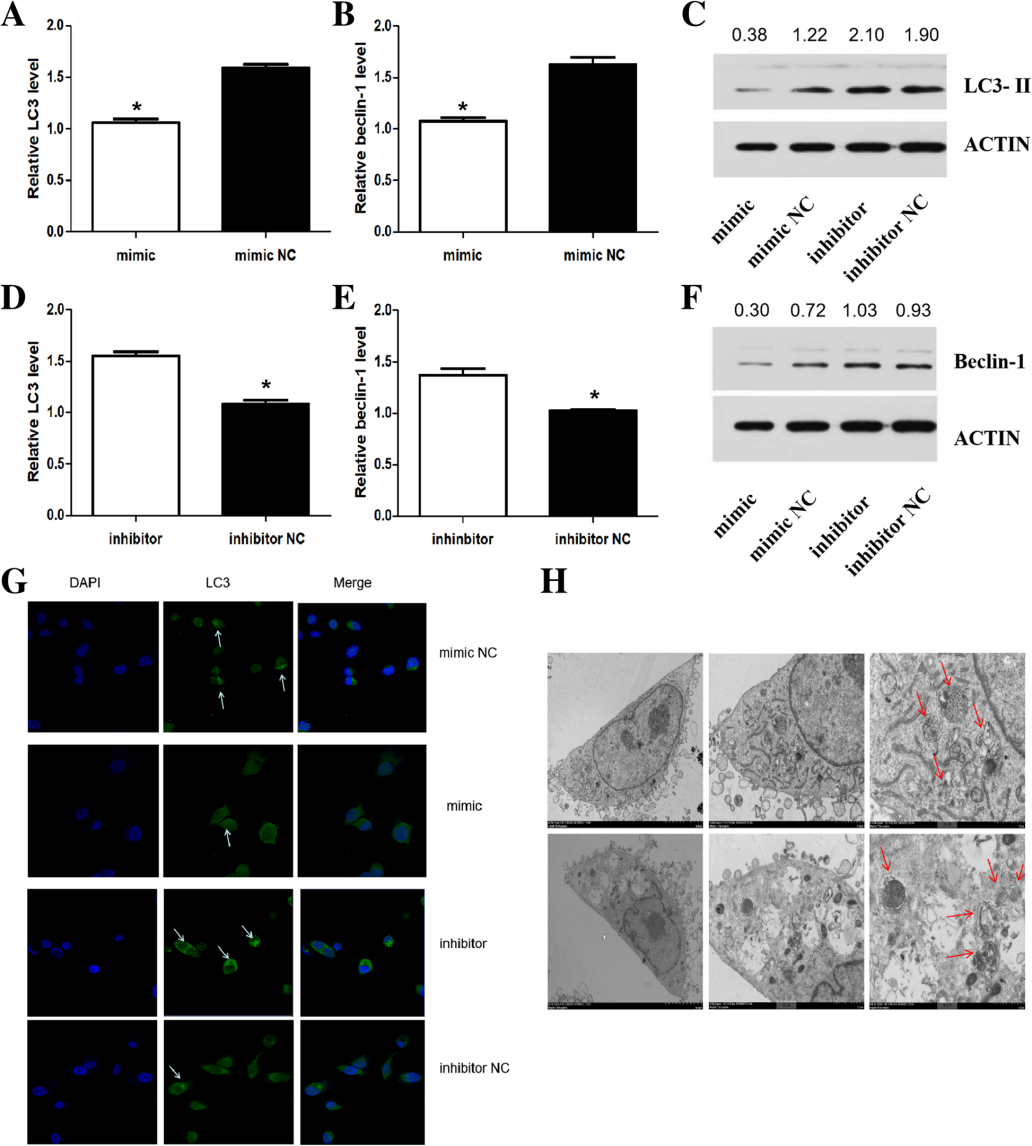
The effect of miR-188-3p overexpression or inhibition on autophagy. **A-B** LC3 (**A**) and Beclin-1 (**B**) gene expression in the miR-188-3p mimic group was lower than the LC3 and Beclin-1 gene expression in the miR-188-3p mimic NC group. **C** LC3 II protein in the miR-188-3p mimic group was decreased. **D-E** LC3 (**D**) and Beclin-1 (**E**) gene expression in the miR-188-3p inhibitor group was higher than the LC3 and Beclin-1 gene expression in the miR-188-3p mimic NC group. **F** Beclin-1 protein in the miR-188-3p mimic group was decreased. **G** The degree of punctate aggregation of LC3 decreased in the miR-188-3p mimic group and increased in the miR-188-3p inhibitor group. DAPI fluorescence is shown in blue, and LC3 II fluorescence is shown in green. White arrows mark LC3 II fluorescence. **H** The number of autophagosomes in the miR-188-3p mimic group was alleviated. Red arrows mark autophagosomes. *Statistically significant difference compared with the control group (*P* < 0.05)

To investigate the effect of miR-188-3p overexpression or inhibition on autophagy, the expression of the autophagy marker genes LC3 (*P* = 0.010) and Beclin-1 (*P* = 0.024) was observed. We found that LC3 and Beclin-1 expression in the miR-188-3p mimic group was dramatically alleviated compared with the LC3 and Beclin-1 expression in the miR-188-3p mimic NC group (Fig. 4A-C, 4F). In addition, the levels of the two were effectively higher in the miR-188-3p inhibitor group (*P* < 0.05) (Fig. 4C-F).

Subsequently, the location of LC3 was detected by immunofluorescence, located mainlyin the cytoplasm. As expected, the degree of punctate aggregation of LC3 decreased in the miR-188-3p mimic group, while the degree of punctate aggregation of LC3 of the miR-188-3p inhibitor group increased. Diamidino-2-phenylindole (DAPI) fluorescence is shown in blue, and LC3 fluorescence is shown in green (Fig. 4G).

Using transmission electron microscopy, we found that the number of autophagosomes in the miR-188-3p mimic group was alleviated. Autophagosomes were distinguished from other cellular structures within the cell by a double-layer or multilayer membrane and contained damaged or ageing organelles and cytoplasmic content (Fig. 4H).

## Discussion

miRNAs regulate gene posttranslation by binding to specific mRNA targets and participating in various physiological and pathological regulatory processes [10]. Preliminary experiments ascertained that miRNA expression in spermatogenic cells changes at different stages, which indicates that miRNAs contribute to spermatogenic cell development and spermatogenesis. Recent studies confirmed that miRNAs, as key mediators of human and animal testicular development and spermatogenesis, are involved in early male germ cell proliferation and late spermatogenesis to form functional sperm^3^. Since spermatogenesis is a complex differentiation process, NOA may be caused by abnormal gene expression at any stage during spermatogenesis [11]. Accumulating evidence has confirmed that miR-188-3p is abundantly detected in spermatogonia and spermatocytes in human testicular tissue and during the meiotic cell cycle of mouse cells [4]. Moreover, our previous research data supported that miR-188-3p expression in NOA testicular tissue was significantly reduced, indicating that dysregulated miR-188-3p expression may contribute to the occurrence of NOA. In this experiment, a total of 109 differentially expressed mRNAs were identified by high-throughput sequencing analysis of testicular tissues of patients in the two groups, and 5 mRNAs were predicted by bioinformatics analysis to be targets of miR-188-3p. The identified potential binding sites provided a new target for our subsequent study of the effect of miR-188-3p in spermatogenesis.

According to previous studies, ATG7, as an autophagy gene and an E1-like activating enzyme, is essential for the autophagy coupling system and autophagosome formation. ATG7 can simultaneously control the number and size of autophagosomes [12]. Studies have also shown that ATG7 is crucial for the formation of the ATG5/ATG12/ATG16 L complex and LC3 complex and contributes to the regulation of the autophagy pathway [13]. In animal experiments, Wang et al. [14] found that male mice with ATG7 mutations exhibited complete infertility and abnormal acrosome formation, consistent with human round sperm disease, a severe fertility disorder characterized by abnormal round head sperm. Subsequently, the expression of ATG7 and the levels of autophagy and apoptosis were found to be increased in males with infertility due to varicocele, which may be related to the elimination of numerous abnormal sperm and the response to heat and oxidative stress [15]. Our results showed that ATG7 was considerably enhanced in NOA testicular samples and was expressed in spermatogenic cells at all levels, suggesting that ATG7 may participate in the development of NOA through high levels of autophagy.

Previous studies have shown that the expression phenotype of testicular tissue in NOA patients is characterized by the loss of normal spermatogenesis, and some upregulated mRNA genes are caused by the downregulation of some miRNAs [16]. Similarly, another study confirmed that autophagy promoting factor (APF) regulates autophagy cell death by targeting miR-188-3p and ATG7 [17]. In addition, proteins related to chromosome structure (such as histones and dislocation repair proteins) may have binding sites with miR-188-3p, and these proteins are crucial for spermatogenesis [18]. miR-188-3p was also proven to be the target of polypyrimidine bundle binding protein (PTBP) 2 in mouse testes, and PTBP2 binds to the 3'-UTR of germ cell-specific phosphoglycerate kinase 2. Therefore, there may be another potential pathway by which miR-188-3p regulates PTBP2 involvement in spermatogenesis [19]. Our previous study detected that miR-188-3p expression is lower in NOA patients. In this experiment, higher expression of the ATG7 gene was found in the NOA group. In addition, ATG7 was downregulated after miR-188-3p overexpression in the human testicular cancer cell line NTERA-2 and was upregulated after miR-188-3p inhibition. The above results suggest that ATG7 gene expression is impeded by miR-188-3p. However, whether miR-188-3p and ATG7 have a direct targeted regulatory relationship in testicular tissues to affect spermatogenesis has not been reported. We further predicted that the 3'-UTR of ATG7 has potential binding sites with miR-188-3p through bioinformatics. Additionally, the dual luciferase reporter assay results showed that after miR-188-3p overexpression, the luciferase activity of the ATG7 3’-UTR WT group decreased, while the luciferase activity of the mutant ATG7 3’-UTR showed no significant difference. Based on these results, we speculate that miR-188-3p may contribute to autophagy by regulating ATG7 expression, leading to spermatogenesis disorder and then NOA.

Autophagy is a relatively conservative evolutionary mechanism in eukaryotic cells that is important for normal cell development and physiological processes. Autophagy begins with the formation of autophagosomes. In most cases, autophagy is recognized to protect and destroy cells [20]. LC3 is a homologous gene of ATG8 in mammals [21] and has two mutually transformable forms, LC3-I and LC3-II. After induction of autophagy, LC3 is cleaved by the cysteine protease ATG4 to form LC3-I. LC3-I is coupled to the substrate phosphatidylethanolamine (PE) to form the autophagosomal membrane-bound form LC3-II. This process is performed by the E1-like enzyme ATG7, the E2-like enzyme ATG3, and the E3-like enzyme ATG5-ATG12-ATG16 L complex. LC3-II, an important marker molecule for autophagosomes, increases with an increase in autophagosomal membranes [22]. When autophagy is initiated, LC3-I will enzymatically cleave a small piece of polypeptide and transform into LC3-II. Therefore, the autophagy level can be estimated by the LC3-II/I ratio [23]. Wei et al. [24] found that the abnormal increase and decrease in LC3-II levels led to autophagy defects, thereby impairing the spermatogenic function of rat testes and leading to testicular developmental disorders. Beclin-1, as a homologue of ATG6 in mammals, is the core component of the autophagy mechanism [25]. Beclin-1 is phosphorylated by unc-51-like autophagy activating kinase-1 (ULK1) and acts as an overall scaffold for the phosphoinositide 3-kinase (PI3K) complex, promoting protein recruitment to autophagic vesicles. When the complex binds to other regulatory proteins, it selectively participates in different stages of autophagy [26]. In addition, Beclin-1 has an independent mechanism of autophagy and an independent role in autophagy in cancer [27]. In chronic myeloid leukaemia, miR-21 is upregulated. The expression levels of Beclin-1, Vps34, and LC3-II are increased with antimiR-21 treatment, which ultimately leads to increased autophagy [28]. In this study, LC3 protein was found in the cytoplasm of spermatogenic cells at all levels through immunohistochemistry. In addition, the expression of LC3 and Beclin-1 in NOA testicular samples was higher than the expression of LC3 and Beclin-1 in normal tissues. In patients with NOA, the expression of the two was higher, as detected by Western blot. The results indicate that highly expressed LC3 and Beclin-1 may participate in the occurrence of NOA through autophagy.

miRNAs can regulate target gene expression at the chromatin organizational, transcriptional and posttranscriptional levels to participate in cellular autophagy. miRNAs target the 3'-UTR of mRNAs, turn on/off the gene expression of key autophagy factors after transcription, and regulate the promotion of autophagy factors to trigger or inhibit the occurrence and development of cellular autophagy. The interaction between miR-471-5p and autophagy member proteins is known to regulate the clearance of apoptotic germ cells through LC3-related phagocytosis. Transgenic mice expressing miR-471-5p in Sertoli cells (SCs) showed increased germ cell apoptosis and impaired male fertility [29]. In our study, autophagy marker gene expression in NOA testicular tissue was higher than the autophagy marker gene expression in the normal group. Additionally, in cell experiments, LC3 and Beclin-1 expression was lower after miR-188-3p overexpression. After miR-188-3p suppression, LC3 and Beclin-1 were similarly enhanced in the miR-188-3p inhibitor group. The immunofluorescence results showed that after miR-188-3p overexpression, LC3 spot aggregation was elevated in the miR-188-3p mimic group compared with the miR-188-3p mimic NC group, whileLC3 spot aggregation was decreased in the miR-188-3p inhibitor group. Finally, autophagy was further monitored through transmission electron microscopy [30]. The number of autophagosomes in the miR-188-3p mimic group was lower than the number of atophagosomes in the miR-188-3p mimic NC group according to transmission electron microscopy. Our results indicated that miR-188-3p is related to the occurrence of autophagy. In our study, first, the expression of ATG7 in testicular tissues of the NOA group and normal group was confirmed to be elevated by high-throughput sequencing. Notably, Wang et al. [17] also confirmed that miR-188-3p impedes autophagy by regulating ATG7. We further used dual luciferase experiments to verify that miR-188-3p has a mutual regulatory relationship with ATG7 and that ATG7 is the target site for miR-188-3p. There may be downregulation of miR-188-3p in the testicular samples by reducing these two genes. The inhibitory effect of ATG7 in turn leads to an abnormal increase in autophagy levels, which affects the spermatogenesis process.

In summary, the results of our study indicate that miR-188-3p may regulate its target gene ATG7 to participate in autophagy and affect the occurrence and development of NOA, providing a new experimental and theoretical basis for studying autophagy as a potential target for diagnosis and treatment. Combined with our previous research results that miR-188-3p-targeted regulation of MLH1 affects apoptosis and participates in the occurrence of NOA, it will be possible to explore whether there is an autophagy-apoptosis interaction network affecting NOA.

## Supplementary Information

**Additional file 1:** **SupplementaryTable SI. **Primers for qRT - PCR (premier 3.0).

**Additional file 2:** **Supplementary Table SII. **qReal-time PCRreaction solution configuration.

**Additional file 3:** **Supplementary Table SIII.** PCR reaction condition setting.

**Additional file 4:** **SupplementaryTable SIV. **mRNAs with potential binding sites formiR-188-3p.

## Data Availability

All data generated or analysed during this study are included in this published article and its supplementary information files.
