# Supplementary material for: miR-188-3p-targeted regulation of ATG7 affects cell autophagy in patients with nonobstructive azoospermia
Source: Reprod Biol Endocrinol. 2022 Jun 16;20:90. doi: 10.1186/s12958-022-00951-0 (PMC9202134; doi:10.1186/s12958-022-00951-0)
Supplement: Supplementary file 1 — Additional file 1: SupplementaryTable SI. Primers for qRT - PCR (premier 3.0). [file 12958_2022_951_MOESM1_ESM.docx]

**Supplementary Table SI.** Primers for qRT - PCR (premier 3.0)

| Gene name | Primer | Sequences（5^，^-3^，^） |
| --- | --- | --- |
| ATG7 | Forward | 5’-TCCGGGGATTTCTTTCACGG-3’ |
|  | Reverse | 5’-GCTTCATCCAGAGCCGAAGA-3’ |
| LC3 | Forward | 5’-GATGTCCGACTTATTCGAGAGC-3’ |
|  | Reverse | 5’-TTGAGCTGTAAGCGCCTTCTA-3’ |
| Beclin-1 | Forward | 5’-AGCTGCCGTTATACTGTTCTG-3’ |
|  | Reverse | 5’-ACTGCCTCCTGTGTCTTCAATCTT-3’ |
| GAPDH | Forward | 5’-GGTGGTCTCCTCTGACTTCAACA-3’ |
|  | Reverse | 5’-GTTGCTGTAGCCAAATTCGTTGT-3’ |
| miR-188-3p | Forward | 5’-ATTATTGGCTCCCACATGCAGGG-3’ |
|  | Reverse | 5’-ATCCAGTGCAGGGTCCGAGG-3’ |

**Footnotes**

#### GAPDH was used as an internal reference gene.
