# Supplementary material for: miR-188-3p-targeted regulation of ATG7 affects cell autophagy in patients with nonobstructive azoospermia
Source: Reprod Biol Endocrinol. 2022 Jun 16;20:90. doi: 10.1186/s12958-022-00951-0 (PMC9202134; doi:10.1186/s12958-022-00951-0)
Supplement: Supplementary file 2 — Additional file 2: Supplementary Table SII. qReal-time PCRreaction solution configuration. [file 12958_2022_951_MOESM2_ESM.docx]

**Supplementary Table SII.** qReal-time PCR reaction solution configuration

| Reagent | Dosage |
| --- | --- |
| QN SYBRGreen PCR Master Mix (2×) | 5μl |
| PCR Forward primer (20μM) | 0.35μl |
| PCR Reverse primer (20μM) | 0.35μl |
| ROX Reference Dye or Dye II | 0.05μl |
| RT reaction solution (cDNA solution) | 1μl |
| dH_2_O | 3.25μl |
| Total | 10μl |
