# Supplementary material for: miR-188-3p-targeted regulation of ATG7 affects cell autophagy in patients with nonobstructive azoospermia
Source: Reprod Biol Endocrinol. 2022 Jun 16;20:90. doi: 10.1186/s12958-022-00951-0 (PMC9202134; doi:10.1186/s12958-022-00951-0)
Supplement: Supplementary file 3 — Additional file 3: Supplementary Table SIII. PCR reaction condition setting. [file 12958_2022_951_MOESM3_ESM.docx]

**Supplementary Table SIII.** PCR reaction condition setting

| Stage | Cycle | Temperature(℃) | Duration(s) |
| --- | --- | --- | --- |
| holding stage |  | 95 | 120 |
| cycling stage | 40 cycles | 95 | 5 |
|  |  | 60 | 34 |
| melting stage |  | 95 | 15 |
|  |  | 60 | 60 |
|  |  | 95 | 30 |
|  |  | 60 | 15 |
