# Supplementary material for: miR-188-3p-targeted regulation of ATG7 affects cell autophagy in patients with nonobstructive azoospermia
Source: Reprod Biol Endocrinol. 2022 Jun 16;20:90. doi: 10.1186/s12958-022-00951-0 (PMC9202134; doi:10.1186/s12958-022-00951-0)
Supplement: Supplementary file 4 — Additional file 4: SupplementaryTable SIV. mRNAs with potential binding sites formiR-188-3p. [file 12958_2022_951_MOESM4_ESM.docx]

**Supplementary Table SIV.** mRNAs with potential binding sites for miR-188-3p

| Gene name | Regulation | Multiple of difference |
| --- | --- | --- |
| IGFBP4 | upregulated | 2.77 |
| MPEG1 | upregulated | 3.83 |
| NEGR1 | upregulated | 2.64 |
| ATG7 | upregulated | 2.58 |
| ISY1-RAB43 | downregulated | 2.35 |
